# Treatment of acute uncomplicated diverticulitis without antibiotics: risk factors for treatment failure

**DOI:** 10.1007/s00384-018-3055-1

**Published:** 2018-04-21

**Authors:** HE Bolkenstein, WA Draaisma, BJM van de Wall, ECJ Consten, IAMJ Broeders

**Affiliations:** 1 Department of Surgery, Meander Medical Centre, 3800 BM Amersfoort, The Netherlands; 2 Robotics and Mechatronics, Faculty of Electrical Engineering, University of Twente, 7500 AE Enschede, The Netherlands; 3 Department of Surgery, Jeroen Bosch Hospital, Den Bosch, The Netherlands; 4 Department of Surgery, Diakonessenhuis, Utrecht, The Netherlands

**Keywords:** Diverticulitis, Complications, Treatment failure, Risk factors, Antibiotic treatment

## Abstract

**Purpose:**

Conservative treatment strategy without antibiotics in patients with uncomplicated diverticulitis (UD) has proven to be safe. The aim of the current study is to assess the clinical course of UD patients who were initially treated without antibiotics and to identify risk factors for treatment failure.

**Methods:**

A retrospective cohort study was performed including all patients with a CT-proven episode of UD (defined as modified Hinchey 1A). Only non-immunocompromised patients who presented without signs of sepsis were included. Patients that received antibiotics within 24 h after or 2 weeks prior to presentation were excluded from analysis. Patient characteristics, clinical signs, and laboratory parameters were collected. Treatment failure was defined as (re)admittance, mortality, complications (perforation, abscess, colonic obstruction, urinary tract infection, pneumonia) or need for antibiotics, operative intervention, or percutaneous abscess drainage within 30 days after initial presentation. Multivariable logistic regression analyses were used to quantify which variables are independently related to treatment failure.

**Results:**

Between January 2005 and January 2017, 751 patients presented at the emergency department with a CT-proven UD. Of these, 186 (25%) patients were excluded from analysis because of antibiotic treatment. A total of 565 patients with UD were included. Forty-six (8%) patients experienced treatment failure. In the multivariable analysis, a high CRP level (> 170 mg/L) was a significant predictive factor for treatment failure.

**Conclusion:**

UD patients with a CRP level > 170 mg/L are at higher risk for non-antibiotic treatment failure. Clinical physicians should take this finding in consideration when selecting patients for non-antibiotic treatment.

## Introduction

Diverticulitis is a common and costly disease. It is now ranked as the third most common gastrointestinal discharge diagnosis and an estimate of 2.1 billion dollars per year are spent on inpatient costs in the USA [1]. Most patients (75%) have uncomplicated diverticulitis (UD), which is defined by the absence of abscess, perforation, fistula, or bleeding [2]. Traditionally, UD was treated in hospital with antibiotics and bowel rest [3, 4]. In the past years, evidence has been presented which justifies a more liberal approach. Two recent randomized clinical trials comparing antibiotic treatment with non-antibiotic treatment in UD patients showed no beneficial effect of antibiotic treatment in this patient group [5, 6]. Moreover, recent studies have provided strong evidence that the outpatient treatment of UD patients is safe and effective even without oral antibiotics [7–12]. However, these studies do report treatment failure rates of 3–24% and due to high risk of selection and detection bias the results are less applicable to daily practice [10]. The question remains which UD patients are eligible for non-antibiotic treatment and which UD patients are more susceptible for a complicated course (treatment failure) and should therefore receive antibiotic treatment and closer surveillance. Few studies have investigated clinical risk factors for treatment failure in patients with UD. In the few studies that are available, all patients received antibiotics [8, 9]. The aim of the current study is to assess the clinical course of UD patients who were initially treated without antibiotics and to identify risk factors for treatment failure in this patient group.

## Methods

### Study design

A retrospective cohort study was performed in the Meander Medical Centre, the Netherlands. Data were collected between January 2005 and January 2017. The study was approved by the local Institutional Review Board.

### Study population

A diagnostic specific code was used to identify all adult (≥ 18 years of age) patients presenting with a first episode of acute UD in the emergency department. The diagnosis had to be proven by a computed tomography (CT) scan. UD was defined as the absence of perforation (extravasation of contrast on CT), abscess, bleeding, or signs of peritonitis, which corresponds to the modified Hinchey classification 1A [13–15]. Only non-immunocompromised patients who presented without signs of sepsis were included in the study. Patients that received antibiotics within 24 h after or 2 weeks prior to presentation were excluded from analysis.

### Outcome measures

Patient characteristics, clinical signs and symptoms, American Society of Anesthesiologists (ASA) Physical Status classification scores, physical examination, laboratory parameters, CT-findings, and treatment data (e.g., surgery, abscess drainage, antibiotic treatment, watchful waiting) were collected from the hospital records. Treatment failure was defined as (re)admittance, mortality, complications (perforation, abscess, colonic obstruction, urinary tract infection, pneumonia) or need for antibiotic treatment, operative intervention, or percutaneous abscess drainage within 30 days after initial presentation.

### Statistical analysis

Multiple imputation techniques were used to impute missing data points in order to avoid selection bias. Data were assumed to be missing at random. All reported results are based on the imputed data, where the estimates of interests at the final computational step were combined across the imputed datasets using Rubin’s rules [16]. Descriptive statistics were provided of all variables. Continuous variables are presented as means (with standard deviation (SD)) or medians (with inter quartile range (IQR)) according to their distribution. For the categorical variables, the counts and percentages are presented. In the initial analysis, the differences in patient characteristics, signs, symptoms, and additional tests between patients with and without treatment failure were assessed. Univariable logistic regression analyses were used to calculate the rude odds ratios (OR) with 95% confidence interval (CI) of the independent predictors. These analyses were used to quantify which (combination of) variables are independently related to treatment failure. Inclusion of the relevant diagnostic items in the multivariable model were based on clinical knowledge and *p* values (*p* value < 0.10). To correct for a possible treatment effect, hospitalization (hospital admittance within 24 h after presentation) was included in the multivariable regression model. All analyses were performed using the statistical software package SPSS 24.0 (IBM Corporation, New York, USA).

## Results

### Patient demographics and initial treatment strategy

**Fig. 1:**
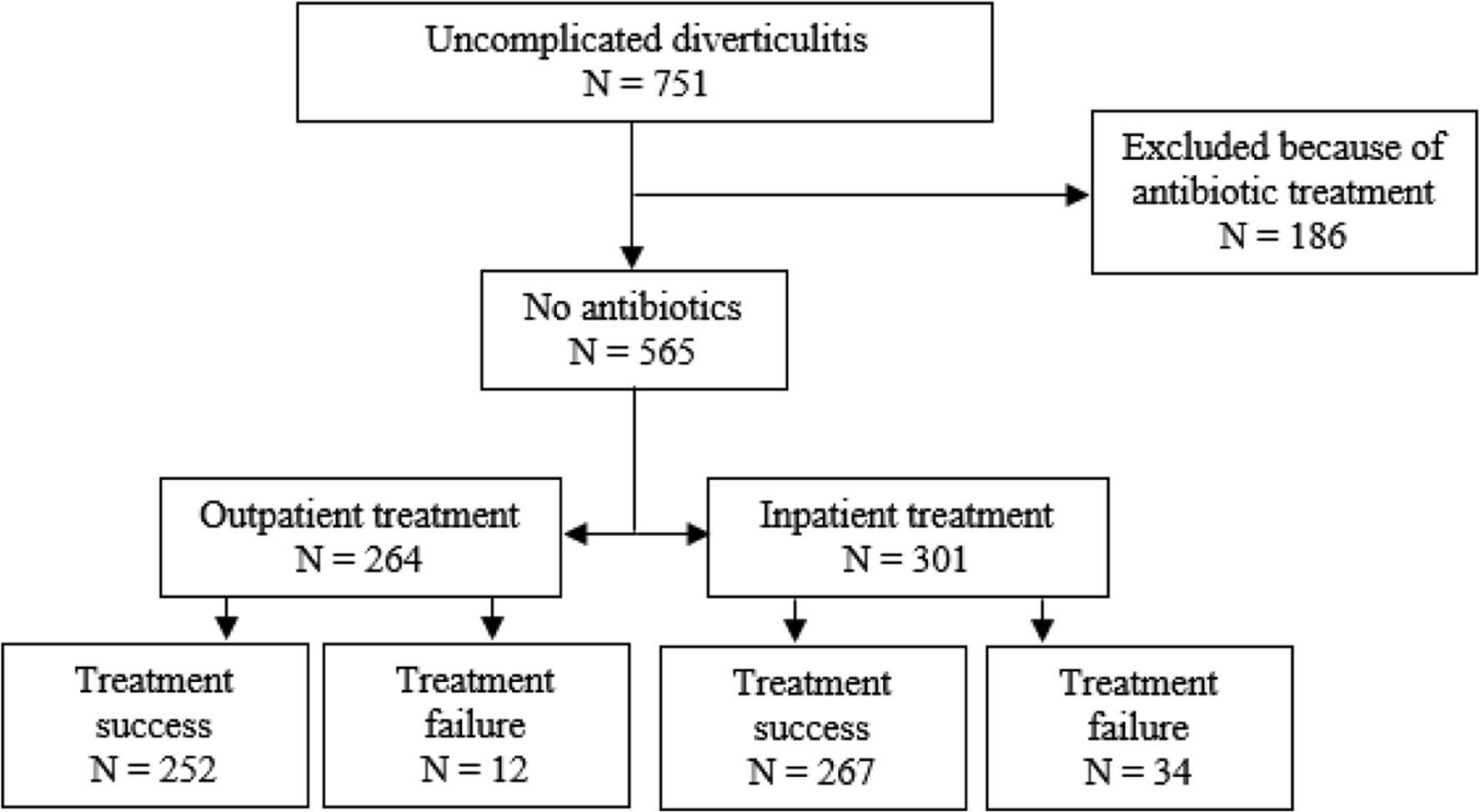
Patient flow throughout study

Figure 1 shows the patient flow throughout the study. Between January 2005 and January 2017, 751 patients presented at the emergency department with a CT-proven Hinchey 1A diverticulitis [13–15]. Of these, 186 (25%) patients received antibiotics within 24 h after or 2 weeks prior to presentation and were excluded from analysis. A total of 565 patients with CT-proven Hinchey 1A diverticulitis [13–15] were included. Patient demographics are shown in Table 1. The average age was 58 (SD 13) years, and 60% of the patients were female. Three hundred one patients (53%) were admitted to the hospital within 24 h after presentation. Patients who were admitted to the hospital within 24 h after presentation presented more often with nausea (43 vs 34%), vomiting (12 vs 5%), and active muscle resistance at physical examination (19 vs 12%) compared to patients who were treated as outpatients. Temperature (mean 37.4 vs 37.2 °C), leucocytes (mean 12.3 × 10^9^/L vs 11.3 × 10^9^/L), and C-reactive protein (CRP) level (mean 104 vs 84 mg/L) were also higher at presentation in patients admitted to the hospital.

**Table 1:** Characteristics of UD patients treated without antibiotics

Variable	All patients *N* = 565	Outpatient treatment *N* = 264 (47%)	Inpatient treatment^1^ *N* = 301 (53%)	*p* value
Patient history				
Gender (*N* (%) female)	338 (60)	162 (61)	176 (58)	0.48^2^
Age in years; mean (SD)	58 (13)	57 (12)	59 (13)	0.08^4^
ASA score > 2, *N* (%)	53 (9)	22 (8)	31 (10)	0.47^2^
Duration of symptoms in days; median (IQR)	3 (1–6)	3 (1–5)	3 (1–6)	0.73^5^
Nausea, *N* (%)	221 (39)	91 (34)	130 (43)	0.03^2^
Vomiting, *N* (%)	46 (8)	12 (5)	35 (12)	< 0.01^2^
Generalized abdominal pain, *N* (%)	34 (6)	12 (5)	22 (7)	0.17^2^
Feces, *N* (%)				0.14^23^
Diarrhea	77 (14)	36 (14)	41 (14)	
Obstipation	85 (15)	30 (11)	55 (18)	
Alternating	42 (7)	19 (7)	23 (8)	
Rectal blood loss, *N* (%)	39 (7)	14 (5)	25 (8)	0.12^2^
Physical examination				
Rebound tenderness, *N* (%)	193 (34)	90 (34)	103 (34)	0.86^2^
Active muscle, *N* resistance (%)	90 (16)	32 (12)	57 (19)	0.03^2^
Temperature in Celsius, mean (SD)	37.3 (0.7)	37.2 (0.6)	37.4 (0.7)	< 0.01^4^
Laboratory parameters				
CRP mg/L, mean (SD)	94 (68)	84 (55)	104 (77)	< 0.01^4^
Leucocytes ×10^9^/L, mean (SD)	11.8 (3.5)	11.3 (3.1)	12.3 (3.7)	< 0.01^4^
CT findings				
Pericolic free air, *N* (%)	41 (7)	13 (5)	28 (9)	0.04^2^

*Abbreviations*: UD uncomplicated diverticulitis defined as Hinchey 1A, ASA American Society of Anesthesiologists, SD standard deviation, IQR inter quartile range, OR odds ratio, CI confidence interval, CRP C-reactive protein

^1^Hospital admittance within 24 h after presentation

^2^Chi-square test

^3^Fisher exact test

^4^Independent *T* test

^5^Mann-Whitney *U* test

### Missing data

All candidate predictors had missing data except for age, gender, and ASA classification. The percentage of missing data per predictor was between 1% (pericolic free air on CT) and 7% (temperature, nausea, and vomiting). In total, 304 (3%) data items were imputed. Four hundred sixty-one (82%) patients had a complete dataset for all candidate predictors.

### Treatment failure

In total, 46 (8%) patients experienced treatment failure of which 34 patients were admitted to the hospital within 24 h after presentation. Twelve patients were initially treated as outpatients but were admitted to the hospital because of complications or severe complaints. Seventeen patients were readmitted to the hospital within 30 days after initial presentation because of complications or severe complaints. Eighteen patients developed complications: abscess (*n* = 5), perforation (*n* = 8), urinary tract infection (*n* = 2), fistula (*n* = 1), pneumonia (*n* = 1), and colonic obstruction (*n* = 1). Twenty-six patients needed antibiotic treatment due to complications or deterioration of disease. Two patients needed percutaneous abscess drainage. Fourteen patients were operated on within 30 days after presentation of which ten patients were operated in an emergency setting. Indications for operation were perforation (*n* = 8), fistula (*n* = 1), stenosis (*n* = 4), and progression of disease (*n* = 1). Three patients died: one due to perforation and two deaths were not diverticulitis related but died from underlying illness (heart failure and acute myocardial ischemia).

### Risk factors for treatment failure

Table 2 shows the univariable analysis of all candidate predictors. The following variables were included in the multivariable analysis; age, gender, (absence of) rebound tenderness, CRP level, and hospitalization. The results of the multivariable analysis are shown in Table 3. One clinical variable remained as a statistically significant (*p* < 0.05) predictor for treatment failure. Higher CRP (mg/L) level was positively related with treatment failure. Thresholds were introduced for CRP level to further illustrate the predictive value of this parameter. A CRP level of > 170 mg/L yielded the highest diagnostic accuracy with a positive predictive value (PPV) of 17% (95% CI 8–29), a negative predictive value (NPV) of 93% (95% CI 90–95), a sensitivity of 20% (95% CI 9–34), and a specificity of 91% (95% CI 89–94) at a 10% risk for treatment failure [17]. A subgroup analysis of only those patients with complete datasets showed similar results (data not shown).

**Table 2:** Distribution and association of individual predictors with treatment failure

	Treatment success *N* = 519 (92%)	Treatment failure *N* = 46 (8%)		
Diagnostic variable^1^	Frequency (%)^3^	Frequency (%)	*p* value	OR (95% CI)
Patient history				
Female gender, *N* (%)	305 (59)	33 (72)	0.09^2^	1.78 (0.92–3.46)
Age in years, mean (SD)	58 (13)	63 (12)	0.02^4^	1.03 (1.01–1.06)
ASA score > 2, *N* (%)	45 (9)	8 (17)	0.05^2^	2.22 (0.98–5.04)
Duration of complaints in days, median (IQR)	3 (1–5)	3 (1–10)	0.34^5^	1.03 (1.002–1.05)
Nausea, *N* (%)	203 (39)	18 (39)	0.82^2^	1.04 (0.55–1.97)
Vomiting, *N* (%)	40 (8)	7 (15)	0.13^2^	2.01 (0.79–5.11)
Generalized abdominal pain, *N* (%)	30 (6)	4 (9)	0.43^2^	1.55 (0.52–4.62)
Change in bowel habit			0.65^3^	
Diarrhea, *N* (%)	69 (13)	8 (17)		1.43 (0.62–3.28)
Obstipation, *N* (%)	79 (15)	6 (13)		0.85 (0.32–2.24)
Alternating, *N* (%)	37 (7)	4 (10)		1.41 (0.47–4.17)
Rectal blood loss, *N* (%)	33 (6)	5 (11)	0.18^2^	1.93 (0.72–5.17)
Physical examination				
No rebound tenderness, *N* (%)	336 (65)	36 (78)	0.06^2^	2.02 (0.94–4.33)
Active muscle resistance, *N* (%)	81 (16)	9 (20)	0.53^2^	1.28 (0.58–2.82)
Temperature in Celsius, mean (SD)	37.3 (0.7)	37.3 (0.6)	0.76^4^	0.93 (0.60–1.46)
Blood tests				
CRP (mg/L), mean (SD)	92 (66)	121 (85)	0.01^4^	1.01 (1.001–1.01)
Leucocytes(10^9^/L), mean (SD)	11.8 (3.4)	12.3 (3.6)	0.37^4^	1.04 (0.96–1.13)
CRP findings				
Pericolic free air, *N* (%)	35 (7)	6 (13)	0.12^2^	2.06 (0.82–5.19)
Treatment				
Hospital admittance, *N* (%)^6^	267 (51)	34 (74)	< 0.01^2^	2.67 (1.35–5.28)

All results in this table are results of multiple imputation and based on univariable logistic regression

*Abbreviations*: ASA American Society of Anesthesiologists, SD standard deviation, IQR inter quartile range, OR odds ratio, CI confidence interval, CRP C-reactive protein

^1^Variables are coded such that the reported category indicate a higher risk of treatment failure

^2^Chi-square test

^3^Fisher exact test

^4^Independent *T* test

^5^Mann-Whitney *U* test

^6^Within 24 h after presentation

**Table 3:** Multivariable analysis of factors associated with treatment failure

Variable	*β* coefficient^1^	Adjusted OR	95% CI	*p* value
Female gender	0.60	1.83	0.92–3.65	0.09
Age	0.02	1.02	0.99–1.04	0.24
ASA score > 2	0.63	1.88	0.78–4.53	0.16
No rebound tenderness	0.72	2.05	0.95–4.43	0.07
CRP (mg/L)	0.01	1.01	1.001–1.01	0.02
Hospitalization^2^	0.89	2.44	1.21–4.90	0.01

All results in this table are results of multiple imputation and analyses are based on multivariable logistic regression analysis, corrected for hospitalization

*Abbreviations*: OR odds ratio, CI confidence interval, ASA American Society of Anesthesiologists, CRP C-reactive protein

^1^*β* coefficients are expressed per 1 unit increase for the continuous variables and for the condition present in dichotomous variables

^2^Within 24 h after presentation

### Patients excluded from analysis

There were 186 UD patients who received antibiotics within 24 h after or 2 weeks prior to presentation. Of these, 27 (15%) patients experienced treatment failure. Patients who received antibiotics generally presented with higher inflammation parameters (temperature (mean 37.8 °C (SD 0.9) vs 37.4 °C (SD 0.7), *p* < 0.01), CRP level (mean 130 mg/L (SD 92) vs 94 mg/L (SD 86), *p* < 0.01), and leucocyte count (mean 12.8 × 10^9^/L (SD 4.5) vs 11.9 × 10^9^/L (SD 3.4), *p* < 0.01)) and a higher ASA score (15% > ASA 2 vs 9% > ASA 2, (*p* = 0.02)) compared to patients who were treated without antibiotics. A multivariable analysis including all 751 UD patients (non-antibiotic and antibiotic treatment) showed that a high ASA score (> 2) and higher CRP level were significant (*p* < 0.05) predictors for treatment failure. When corrected for hospitalization and antibiotic treatment, these predictors remained statistically significant (data not shown).

## Discussion

This study assessed the implementation of non-antibiotic treatment in UD patients and identified risk factors for treatment failure in this patient group. The majority (75%) of patients presenting with UD were initially treated without antibiotics, and treatment failure was seen in 8% of these patients. Moreover, only 12 patients (2%) had severe complications requiring invasive interventions such as percutaneous abscess drainage (*n* = 2) or emergency surgery (*n* = 10). This shows that the implementation of a more conservative approach without antibiotics has been successful. Patients with a high CRP level (> 170 mg/L) were at significantly higher risk for treatment failure.

Hjern et al. [18] was the first to describe non-antibiotic treatment in UD patients and concluded that non-antibiotic treatment is feasible in a selected group of patients. This finding was recently supported by the results of two randomized clinical trials comparing antibiotic treatment with non-antibiotic treatment in UD patients [5, 6]. Current guidelines on treatment of UD are however still ambiguous when it comes to antibiotic treatment. Guidelines published by the American Society of Colon and Rectal Surgeons (ASCRS) in 2014 still recommend antibiotics as part of conservative treatment [19]. The guideline of the World Society of Emergency Surgery (WSES) in 2016 advises to avoid antibiotic therapy in non-immunocompromised UD patients without systemic signs of infection [20]. The guideline of the Dutch association of surgery (NVvH) on acute diverticulitis recommends to start antibiotic treatment in patients with a temperature of > 38.5 °C, signs of sepsis, and deteriorating symptoms, immunocompromised patients, and patients on non-steroidal anti-inflammatory drugs [21]. In the present study, we found no supporting evidence for these criteria. Only CRP level remained as a significant predictor in the multivariable analysis, and temperature was not a significant predictor for treatment failure (*p* = 0.76). Immunocompromised patients and patients presenting with signs of sepsis were not included in the present study. As sepsis is associated with high morbidity and mortality and immunosuppression can increase the complication rate, it stands to reason that these patients should receive antibiotic treatment [20].

Clinical risk factors for treatment failure in UD patients have been scarcely investigated before. Treatment failure rates of 3–24% are reported, depending on the definition of treatment failure [7–12]. Reported risk factors for treatment failure are female gender, free fluid or free air around the colon on CT scan, comorbidity (Ambrosetti score > 3), and an ER admission time between midnight and 6 AM [8, 9]. These studies are however hampered by the fact that all patients received antibiotics, which is now considered a redundant treatment strategy. One recent study did analyze the feasibility of non-antibiotic treatment in acute uncomplicated diverticulitis and reported a treatment failure rate of 4%. However, this study did not identify risk factors for treatment failure [22]. To our knowledge, the present study is the first to analyze clinical risk factors in UD patients treated without antibiotics. The retrospective design of this study comes with natural limitations. Fifty-three percent of the patients were directly admitted to the hospital. The decision to admit a patient to the hospital was made by the attending physician based on individual patient characteristics. Patients who were directly admitted to the hospital were at higher risk for treatment failure (*p* < 0.01). This could however be the result of confounding by indication as clinical physicians might sooner be inclined to admit patients who have a predisposition for treatment failure to the hospital. It remains unclear if direct hospital admittance has an influence on the outcome of interest (treatment failure). We chose to include both inpatients and outpatients in our analysis as previous literature has provided strong evidence that in hospital treatment of UD patients does not have a beneficial effect compared to outpatient treatment [7–12]. To correct for a possible treatment effect, we included hospital admittance in the multivariable analysis.

Twenty-five percent of all UD patients presenting at the ER were excluded from the main analysis because they received antibiotic treatment within 24 h after or 2 weeks prior to presentation. These patients generally presented with higher inflammation parameters and a higher ASA score. Apparently, physicians consider these features a reason to start antibiotics. There were more patients in the antibiotic group with treatment failure (15%) compared to the non-antibiotic group (8%). Because this is a retrospective study, there is a high risk of confounding by indication and no conclusions can be made from this comparison. In a separate analysis including the patient group treated with antibiotics, a high ASA score (> 2) and higher CRP level were found to be risk factors for treatment failure. This could explain for the high risk of treatment failure in the antibiotic group since patients with a high ASA score and higher CRP level were more likely to receive antibiotics.

As we still found a non-antibiotic treatment failure rate of 8%, it is pertinent to adequately select patients who are suitable for a non-antibiotic treatment strategy. To resolve this problem, the present study tried to identify clinical risk factors for treatment failure, which can guide the decision whether or not to treat UD patients with antibiotics. Based on our results, we conclude that UD patients with a CRP level > 170 mg/L are at higher risk for treatment failure. Although not significant in the non-antibiotic group, a high ASA score (> 2) could also be a risk factor for treatment failure. Clinical physicians should take these findings in consideration when selecting patients for non-antibiotic treatment.
